# “Loyalty to organizations” or “loyalty to supervisors”? Research on differential leadership and employee loyalty behavior: A perspective of insiders and outsiders

**DOI:** 10.3389/fpsyg.2022.971624

**Published:** 2022-12-22

**Authors:** Honglie Zhang, Linshen Du, Zhou Jiang

**Affiliations:** ^1^School of Business, Yunnan University of Finance and Economics, Kunming, China; ^2^International Business School, Yunnan University of Finance and Economics, Kunming, China

**Keywords:** supervisor developmental feedback, power distance, differential leadership, loyalty to organizations, loyalty to supervisors

## Abstract

Loyalty has always been a hot topic in the field of organizational behavior, which is of great significance to the operation and management of organizations. Extant studies have found that leadership can have a great impact on loyalty. Differential leadership is a common and unique leadership style in Chinese organizations, but the research between differential leadership and loyalty is still lacking. Based on social exchange theory and social learning theory. This study discusses the relationship between differential leadership and loyalty. By using hierarchical regression analysis and bootstrap method. The results show that differential leadership has a positive effect on employees’ organizational loyalty and supervisory loyalty, and the degree of “loyalty to organizations” is generally lower than “loyalty to supervisors”; under differential leadership style, the supervisory loyalty from insiders is much higher than that from outsiders, but the influence and difference of the two groups (insider and outsider) do not have a significant effect on organizational loyalty. The supervisor developmental feedback mediates the relationship between differential leadership and organizational & supervisory loyalty; power distance positively moderates the relationship between supervisor developmental feedback and organizational & supervisory loyalty, and the higher the perceived power distance of employees, the stronger the indirect effect of developmental feedback from supervisors. This study not only enriches the theory of differential leadership but also provides empirical support for understanding the differences in cognitive behavior between insiders and outsiders.

## Introduction

“Loyalty” has always been a hot topic of research in the field of organizational behavior, which embodies the force of the organization members to actively maintain individual and organization, and is of great importance to the organizational management (Huangfu et al., 2013; Tomic et al., 2018). The level of employee loyalty in an organization profoundly affects the core competitiveness of the organization. In the era of knowledge economy, talent, as an important strategic resource for enterprises, is inextricably linked to the operation of the enterprise and the innovation of the organization. Once the enterprise encounters the talent drain, especially for the core employees, it will cause the loss of technology, leakage of secrets, increase in cost and even affect the survival and development of the enterprise (Kot-Radojewska and Timenko, 2018; Stojanovic et al., 2020). Regarding the study of organizational loyalty, Western scholars are mostly based on the contractual idea of freedom and equality, they see loyalty as employees’ identification with the organization, internalization of values and organizational commitment, and consider loyalty and commitment as interchangeable concepts (Wiener, 1982; Darmawan et al., 2020). However, this contractual view of organizational loyalty is different from that view with the Chinese characteristic of “rule of man is superior to rule of law,” and the item of organizational loyalty in Chinese context contains different cultural connotations compared with western organizational commitment (Lin and Cheng, 2017). Fei (1992) writes in his book: “In the western pattern, all members in an organization are equivalent, just as all straws in a bundle are alike. This is quite different from the Chinese pattern. Social relationships in China possess a self-centered quality. Like the ripples formed from a stone thrown into a lake, each circle spreading out from the center becomes more distant and at the same time more insignificant.” The self-centered quality embodies a differential pattern according to the closeness and distance of relationships with others, which is a typical social psychological characteristic of Chinese societies. Therefore, the concept of loyalty developed on the basis of different underlying logic in the western and oriental cultures is significantly different. Specifically, western scholars emphasize the contracted loyalty of individuals toward an organization, but in the context of China, loyalty is not limited to the relationship between individuals and organizations, which can also be reflected as individual loyalty based on hierarchy, intimacy, and differential sequence (Lin and Cheng, 2017). In the traditional Chinese society, there are two kinds of loyalty: Loyalty to the state and the country (public loyalty) and loyalty to the monarch and the emperor (private loyalty). By extending this concept to the study of Chinese organizational loyalty, two paths can be explored: employee loyalty to the organization and employee loyalty to the supervisor.

Current research on loyalty focuses on sustainability actions, organizational justice, and organizational culture (Sarhan et al., 2020; Cachón-Rodríguez et al., 2021; Jang et al., 2021). Differential leadership as an antecedent variable focuses on organizational citizenship behavior. For instance, Wu (2021) explored the relationship between differential leadership and employee creativity by taking golden-mean thinking as a moderating variable. Wang et al. (2018) studied that differential leadership and employee turnover intention. It is a unique and common phenomenon in Chinese organizations, but few scholars have focused on the relationship between differential leadership and employee loyalty, even if there is a complete gap in the research on differential leadership and “loyalty to organizations” and “loyalty to supervisors.”

According to differential leadership theory, leaders divide their employees into insiders and outsiders based on three factors: “Kinship, loyalty and ability” (Li and Li, 2021). On the one hand, there is no doubt that “loyalty” is a quality valued by all supervisors in Chinese society, even more than “ability.” The old adage that “The monarch should treat his subjects with courtesy, and his subordinates should treat the monarch with loyalty,” loyalty without ability (employee) could be seen as a “booster” for organization, while talent without loyalty is more like a “bomb” (Huangfu et al., 2013). That is to say, supervisors differentiate employees by “loyalty” and divide them into “insider” and “outsider.” On the other hand, insider (perceived insider status), in order to maintain a good relationship with the supervisor and gratitude for the supervisor’s partiality and care, will remain absolute loyal to their supervisors, while the outsider (perceived outsider status), in order to achieve the status transformation from outsider to insider, will imitate the behavior of the insider and constantly show loyalty to their supervisors. Therefore, the first objective of this paper is to investigate whether differential leadership has an effect on employee loyalty in the two-way interaction between supervisors and employees, whether such loyalty is “loyalty to organizations” or “loyalty to supervisors,” and to clarify the difference. Furthermore, the core feature of differential leadership is to divide employees into insiders and outsiders and treat them differently (Wu et al., 2021), but few scholars explore whether differential leadership influences employees’ cognitive behaviors from a perspective of insider and outsider. Based on this, the second objective is to investigate whether differential leadership can cause differences in employee loyalty between insiders and outsiders and the extent of such differences.

The differential leadership treats insiders and outsiders differently, which inevitably leads to different reflections on employees’ psychology and behavior. So, what is the process mechanism of this influence? Scholars such as Jiang and Chang (2010) specifically defined the connotation of differential leadership as three dimensions: communication and care, tolerance, and promotion and rewards from these three dimensions, it can be found that differential leadership not only provides employees with richer material rewards and more promotional opportunities, but also establishes deeper emotional communication with them, such as giving them full trust or being more tolerant when they make mistakes. All kinds of partiality from differential leadership fall under the category of supervisor developmental feedback, which has been confirmed by a large number of previous studies as an important variable in predicting individual behavior and often plays a mediating role in studying organizational citizenship behavior (Yao and Fu, 2019; Bak, 2020; Fang et al., 2021; Hamzah et al., 2021). Therefore, the third objective of this paper is to investigate whether supervisor developmental feedback can play a mediating role between differential leadership and employee loyalty and to clarify its process mechanism.

In addition, the study of employee loyalty behavior in the Chinese context cannot ignore the cultural value orientation of the employee (Farh et al., 2007; Zheng et al., 2018). As a typical country with high power distance, China implements a strict subordinate relationship between superiors and subordinates, and regards obedience and loyalty to superiors as the obligations of subordinates (Kirkman et al., 2009; Lee and Lee, 2018). It can be inferred that employee loyalty and differential leadership are also affected by power distance. However, few domestic scholars research this topic. Therefore, the fourth objective of this paper is to use employee power distance as a moderator variable to investigate its moderating role between differential leadership and employee loyalty, and to clarify the boundary conditions for differential leadership to affect employee loyalty.

## Theory and hypotheses

### Differential leadership and employee loyalty

Fei (1992) pointed out in his book of *From the Soil: the foundations of Chinese society*. That the relationship (guanxi) structure of Chinese society shows a self-centered pattern of differential treatment based on the closeness and remoteness of relationships with others, which echoes the “differential and order of human relationships (ren lun)” mentioned in the *Book of Rites*, the “differential” means “intimacy” (qin qin), which refers to the horizontal relationship of closeness and remoteness based on blood or marriage, such as the relationships (husband and wife, older and younger brothers); “order” means “respect” (zun zun), which reflects the vertical relationship of superiority and inferiority based on rights and obligations, such as the relationship (monarch and subject). As a typical social psychological characteristic of Chinese society, the differential pattern exists not only embodies the macroscopic social aspect, but also the middle organizational aspect and the microcosmic interpersonal aspect (Pang and Ma, 2019), differential leadership would consciously divide employees into insiders and outsiders (Outsiders refer to employees who only maintain formal working relationships with their supervisors, not beyond the contractual relationship, and the exchange quality between them is low; while insiders not only maintain exchange relationships with their supervisors at work, but also maintain emotional exchange relationships, that is, supervisors tend to give insiders more partiality and care in terms of salary, working performance and job promotion, etc., even more trust and tolerance) on the basis of kinship, loyalty, and ability, and give the insider partiality and care in work and life. However, “kinship “, as an antecedent factor, cannot be acquired later in life, therefore, loyalty and ability become the main basis for the supervisor to divide employees into insiders and outsiders (Robinson et al., 2008). The function of loyalty is more like the “threshold” for employees to become a insider, and ability is the screening condition for supervisor to select the insider. In the context of differential pattern, the core of the “relationship” (superior and subordinate) in the organization is “loyalty,” exactly as Huangfu et al. (2013) classified employees into eight categories and believed that loyalty without ability (employee) could be seen as a “booster” for organization, while ability without loyalty is more like a “bomb.” Therefore, to some extent, loyalty is more important than ability in Chinese society.

Under the differential pattern, there is a tripartite interaction mechanism in the formation of behavioral loyalty. From the supervisor’s point of view, they will only give partiality and care to employees (insiders) from the employee’s point of view, the supervisor has the power to control important resources in the organization, which is extremely important to employee’s career development (Xu et al., 2022). According to social exchange theory, interpersonal communication is a process of resource exchange, reflecting in material resources and emotional resources. In daily work and life, both material satisfaction and emotional respect are indispensable. In the interaction of two parties, in order to maintain the stability of the exchange relationships, the beneficiary must pay a corresponding return to the provider based on the principle of reciprocity (Cropanzano and Mitchell, 2005). Therefore, when employees (insiders) get the partiality from differential leadership in terms of salary, performance, and promotion, they would give material rewards to leaders in return in order to maintain a reciprocal relationship (Cropanzano and Mitchell, 2005; Yin, 2018), but due to the limitation of personal ability, they usually cannot provide leaders with considerable money or other material resources in return. Because social exchange not only involves material resources but also emotional resources, insiders tend to show a high degree of loyalty, and provide adequate support for the work of the supervisor, in order to reward them. On the other hand, insiders can get more emotional trust and tolerance from differential leaderships, they manage to maintain this high-level emotional exchange relationship, reflecting in meeting the emotional needs of their supervisors in a way that is loyal—after all, loyalty is the utmost respect for employees to their supervisors (Ma et al., 2022).

On the other hand, the identities of “insider” and “outsider” are not static, but even interchangeable under certain conditions (Lu et al., 2022). The differential leadership, while treating the “insiders” with partiality and care, will also have a certain degree of spurring effect on the “outsiders” (Yi, 2014; Obenauer and Langer, 2019). Social learning theory holds that observing others is an important way of learning, in addition to learning through reading, communicating and experiencing. Vicarious reinforcement mechanisms in social learning theory suggest that people consciously imitate those actions leading to positive outcomes through observation (Bandura, 1977). In Organization Behavior Study, social learning theory explains the mechanism of behavioral transmission among members, that is, members will observe the behavior of surrounding members to have positive behavior, and regard them as role models to follow. Learning through observation mainly occurs among employees, because it is highly visible for each employee to observe each other. The partiality from differential leadership is much like a booster for employee’s career development. In order to obtain more developmental feedback from their supervisors, outsiders must become an insiders. In the process of observing the behavior of insiders, outsiders will see the insiders as role models and try to imitate their behavior in order to receive the same treatment or obtain the expected positive results (Bai et al., 2019).

The function of Loyalty is more like the “threshold” for employees to become an insider from an outsider, and meanwhile, loyalty is the first and most easily behavior for outsiders to imitate.

**Figure 1:**
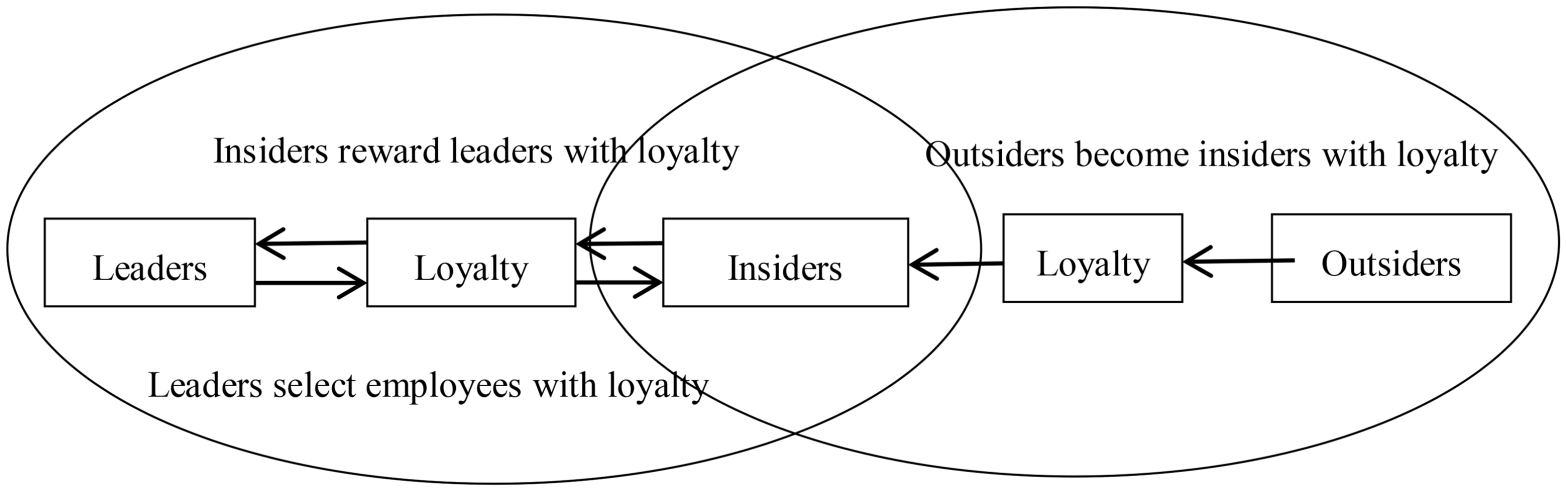
Diagram of the three-way interaction process between differential leadership, insiders and outsiders.

To sum up, differential leadership is the core to decision-making, while providing scarce resources and development convenience for the insider, on the one hand, it strengthens the employee loyalty and identification to the supervisor, and meanwhile, strengthens the cohesiveness of the loyalty atmosphere for the organization. On the other hand, it attracts and promotes outsiders to show their loyalty to the supervisors in order to become an insider, which expands the radiating power of the loyalty atmosphere for the organization. In the process of exchange between the supervisor and the insider, and between the insider and the outsider (as shown in Figure 1), the rule of the circle becomes a kind of psychological contract, which constantly strengthens the employees’ loyalty to the supervisor, but strictly speaking, this loyalty belongs to loyalty to supervisors, but not necessarily loyalty to the organization, or a greater degree of private loyalty and a smaller degree of public loyalty (Tao et al., 2016). As Pye (1992) said, “In Chinese culture, attachment to authority is the best way for employees to gain a sense of security. They can receive protection by exchanging their loyalty and this is a matter of course in their eyes. “By placing the differential pattern in the study of organizational behavior, it can be found that whenever there are different centers of power, a top-down “partisan structure” will emerge (Pye, 1992). It is the result of the presence of different power centers. Although this “partisan structure” is expressed as a “superior-subordinate relationship” with “loyalty”-oriented, since the supervisor is often the concrete embodiment of the organizational image. Therefore, this kind of loyalty is usually regarded as “private loyalty”(loyalty to supervisors) rather than the “public loyalty”(loyalty to organizations). As a result, the following hypothesis is formulated.

**Figure 2:**
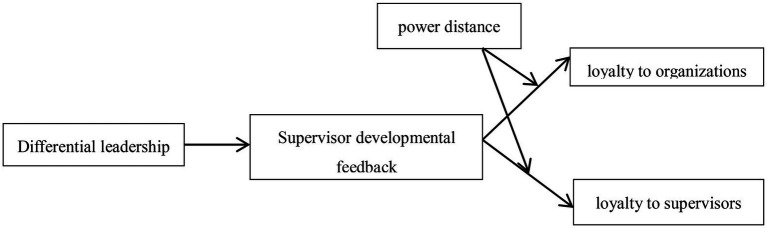
Conceptual model.

*H1:* Differential leadership has a positive effect on employee loyalty, but the effect of “loyalty to supervisors” is greater than that of “loyalty to organizations.”

### The mediating role of supervisor developmental feedback

Developmental feedback from supervisors refers to useful information provided by supervisors to subordinates that can help them learn, develop and improve in the future (Zhou, 2003). It is different from performance feedback. Supervisor developmental feedback not only brings more rewards, but also narrows the psychological distance between superiors and subordinates and strengthens employees’ sense of identity with the organization (Su et al., 2019; Zhang et al., 2019).

Differential leadership treats employees differently through communication, tolerance, and promotion (Jiang and Chang, 2010). The corresponding supervisor developmental feedback includes giving insiders more career development opportunities and emotional support, delegating much more important tasks to them, and being more tolerant when they make mistakes, while these are not available to outsiders. Differential leadership treats employees differently through different levels of developmental feedback from supervisors, and employees who cement a close relationship with supervisors often receive more supervisor developmental feedback. This is because the contributions of employees in the organization are different, and the loyalty to the supervisors is also different. At this point, If leadership treats each employee equally, it will cause employee dissatisfaction (Zhang et al., 2021). Especially in Chinese organizations where the rule of man is prevalent and the power distance is large, it is often natural for supervisors to give more developmental feedback to insiders, and conversely, it is unreasonable to treat them all the same (Jiang and Chang, 2010). Although some scholars argue that differential leadership can cause divisions within employee groups, resulting in a sense of unfairness and exclusion from the bottom of outsiders’ hearts (Chen et al., 2018; Tang et al., 2018) most scholars have demonstrated through empirical studies that supervisors’ partiality and care for the insiders can stimulate the subjective initiative of the outsider to become an insider or cement a closer relationship with supervisors (Huangfu et al., 2013; Dun and Jibao, 2018; Agarwal, 2019; Su and Xiao, 2022). Further, the differential pattern is also in line with the Chinese concept of justice, where intimacy (qin) is in line with “benevolence,” respect (zun) is in line with “righteousness,” and differential treatment is in line with “rite.” Therefore, most of the employees recognize that the supervisor’s partiality and care is a kind of “reasonable fairness” which can reflect on their behavior and improve it to get more new feedback and gain a closer relationship with supervisors (Huangfu et al., 2013). In addition, the amount of supervisor developmental feedback to insiders reflects the importance that supervisors value on their employees, which is more likely to stimulate emotional motivation to make contributions in return and then maintains or even strengthens loyalty to the supervisor, the effect of it is that differential leadership has a positive effect on employee behavior Zhang Z. et al., 2020). Therefore, this study proposes the following hypotheses (Guo et al., 2020; Tang et al., 2020).

*H2:* Supervisor developmental feedback mediates the relationship between differential leadership and organizational loyalty & supervisory loyalty.

### The moderating effect of power distance

Power distance refers to the degree of individuals’ acceptance of the unequal distribution of power in the organization (Lam and Xu, 2019). Its magnitude can be measured by the power distance index (DPI). The PDI of China is 80, which is a high power distance country (Jang et al., 2021). There is hierarchical organization in high power distance countries, employees maintain a strict subordinate relationship with their supervisors and obey authority (Rockstuhl et al., 2020; Jiang et al., 2021; Zhang et al., 2021). They recognize and accept responsibility for the role of obedience and allegiance to the supervisors (Zhang Y. et al., 2020), and if employees have a higher power distance, they are more likely to accept the differential leadership and recognize the partial behavior (Wang and Guan, 2018; Meyers et al., 2019). Power distance is used as a moderator variable to predict the relationship between leadership style and employee behavior (Farh et al., 20,007). In general, employees with a high power distance respond more positively to the supervisor’s behavior (Zhang et al., 2022), especially when the supervisor gives them developmental feedback, they are more likely to feel a strong sense of “gratitude” and return more loyalty. And meanwhile, employees with a high power distance are more likely to recognize the supervisor’s partial behavior and see it as “reasonable unfairness,” so they will not feel any sense of unfairness or diminish their loyalty to their supervisors and the organization. On the contrary, employees with lower power distance believe that all staffs are equal and regard the supervisor developmental feedback as a perfectly normal occurrence (Zhao et al., 2020; Fan et al., 2021). So, the result is that when they receive supervisor developmental feedback, they do not feel too much rewarded from the bottom of their hearts and believe that this is quite universal. And when faced with differential leadership, they may have difficulty understanding such unfair treatment, and even have a serious sense of unfairness, thus they will consciously or unconsciously diminish their loyalty to the organization and supervisors (Hu et al., 2018). As a result, the following hypothesis is proposed in this paper.

*H3:* Power distance positively moderates the relationship between supervisor developmental feedback and organizational & supervisory loyalty. The relationship between supervisor developmental feedback and organizational & supervisory loyalty is stronger in conditions where the level of perceives power distance is high than in conditions where the level of perceives power distance is low.

### Moderated mediating model

As mentioned above, employees with high perceived power distance believe in authority, respect their supervisors, and pay special attention to their action and movement. Therefore, compared with employees with low perceived power distance, employees with high perceived power distance will be influenced a lot when receiving positive supervisor developmental feedback, reflecting in their cognition and behavior. Especially in Chinese culture, “return a favor with a favor” has always been the mainstream value respected by the Chinese nation, so the active feedback from leaders will inspire them to return to leadership and organization with more loyal behaviors; on the contrary, employees with low perceived power distance will not positively respond to developmental feedback from their supervisors, even they might feel disgusted with that unfair treatment from supervisors. Combining the relationships involved in H2 and H3, this paper further infers a moderated mediating model. Hence, we hypothesize the following:

*H4:* Power distance positively moderates the indirect effect of differential leadership on organizational loyalty & supervisory loyalty via supervisor developmental feedback. The indirect effect of differential leadership on organizational loyalty & supervisory loyalty via supervisor developmental feedback is stronger in conditions where the level of Power distance is high than in conditions where the level of power distance is low.

To sum up, the following theoretical research model is constructed, as shown in Figure 2.

## Materials and methods

### Sample and procedure

The sample of this study comes from 16 enterprises in Yunnan, Guizhou, Sichuan, Beijing, Shenzhen, and Shanghai, involving steel, bio-pharmaceutical, textile, food processing, real estate, finance, and IT industries. The survey time is from December 2021 to March next year. In order to reduce the influence of common method deviation, Podsakoff and other suggestions are adopted. Firstly, before the questionnaire is issued, the confidentiality of the questionnaire is emphasized. The questionnaire is promised to be anonymous, and will never reveal personal privacy off (Podsakoff et al., 2003). It is only used for academic research to ensure that the answerer can rest assured and try to restore the true feelings. Secondly, the questionnaire is distributed in two periods, with a time interval of 2 weeks. In the first period, demographic variables, differential leadership, insider identity cognition, superior developmental feedback scale and leadership loyalty and organizational loyalty scale are filled in. After obtaining the enterprise consent, the questionnaire was issued with the assistance of department heads or human resources departments. The questionnaire was all in the form of electronic questionnaire, and 683 and 645 questionnaires were collected twice, respectively. The questionnaire was filled out for less than 120 s, with obvious errors in reverse questions and continuous selection of the same answer. A total of 480 valid questionnaires were obtained, and the questionnaire recovery rate was 74.4%, of which 70.1% were males and 29.9% were females. Age, under 21 years old accounted for 3.6%, 22–31 years old accounted for 27.7%, 32–41 years old accounted for 44.5%, 42–51 years old accounted for 20.4%, 51 years old and above accounted for 3.6%; the proportion of working with direct leadership in less than 1 year accounted for 26.3%, 1–3 years accounted for 27.5%, 3–5 years accounted for 21.2%, more than 5 years accounted for 25.1%; in the current organization working time less than 1 year accounted for 24.8%, 1–3 years accounted for 29.2%, 3–5 years accounted for 19.5%, more than 5 years accounted for 26.5%; in terms of rank, ordinary staff accounted for 40.6%, basic managers accounted for 36.6%, middle managers accounted for 16.4%, and senior managers accounted for 6.4%.

### Measures

The scales used in this paper are authoritative mature scales, with high reliability and validity. The scales used Likert 7-point scoring method, from 1 (completely disagree) to 7 (completely agree).

### Independent variable: Differential leadership

The measurement of this variable adopts the scale developed by Jiang and Chang (2010), with a total of 14 items (Cronbach’s *α* = 0.903). For example, “When encountering conflicting views, I can clearly stand on the side of my superior (leader).”

### Mediator variable: Supervisor developmental feedback

The measurement of this variable adopts the scale developed by Li et al. (2011), which has three items (Cronbach’s *α* = 0.864), such as “My supervisor (leader) provides feedback to me mainly to help me learn and improve.” The Cronbach *α* coefficient of the scale is 0.864.

### Categorical variable: Cognition of insiders’ status

The measurement of this variable adopts the scale developed by Stamper and Masterson (2002) with six items (Cronbach’s *α* = 0.907), such as “My organization gives me a sense of belonging.” Learned from the literature of relevant scholars like Lin and Cheng (2017), taking the mean value of the variable plus or minus a standard deviation as the division standard. The research sample was divided into two samples: the sample of insiders (high cognition of insiders’ status) and outsiders (low cognition of insiders’ status).

### Dependent variable: Loyalty to supervisors

The measurement of Loyalty to Supervisors adopts the scale compiled by Chen et al. (2002), a total of 16 items (Cronbach’s *α* = 0.910). The scale includes five dimensions: internalization of leadership values, identification with leadership, dedication to leadership, additional efforts to leadership, and attachment to leadership.

### Dependent variable: Loyalty to organizations

The measurement of this variable adopts the scale developed by Jiang and Zhang (2010), with a total of 21 items (Cronbach’s *α* = 0.933), including eight dimensions: internalization obligations, identification with the company, sacrifice for the public, advice and suggestions, assistance to colleagues, active participation, maintenance of public interests, and cooperation and compliance.

### Moderator variable: Power distance

The measurement of this variable adopts the scale developed by Farh et al. (2007), with a total of six items (Cronbach’s *α* = 0.831), such as: “leadership decisions do not need to consult the views of employees.”

### Control variables

We controlled for an assortment of variables, including age, gender, rank, working time with direct leaders, and current organizational working time are used as control variables.

## Data analysis and results

### Distinguish validity test and common method deviation test

In this paper, confirmatory factor analysis is used to test the discriminant validity of differential leadership, superior developmental feedback, Power distance, organizational loyalty, and leadership loyalty. The results are shown in Table 1. The fitness index of the five-factor model (*χ*^2^/df = 2.918, TLI = 0.840, CFI = 0.852, GFI = 0.841, NFI = 0.847, RMSEA = 0.068, ∆χ^2^ = 265.426，∆ df = 4, *p* < 0.001) basically meets the requirements. The factor loadings of all indicators of their constructs ranging from 0.517 to 0.891 are significant at the 0.001 level. Compared to the four-factor model (*χ*^2^/df = 3.152, TLI = 0.820, CFI = 0.834, GFI = 0.816, NFI = 0.823, RMSEA = 0.072), three-factor model (*χ*^2^/df = 3.841, TLI = 0.762, CFI = 0.787, GFI = 0.759, NFI = 0.777, RMSEA = 0.083), two-factor model (*χ*^2^/df = 4.408, TLI = 0.715, CFI = 0.736, GFI = 0.711, NFI = 0.726, RMSEA = 0.091), and single-factor model (*χ*^2^/df = 5.174, TLI = 0.651, CFI = 0.650, GFI = 0.646, NFI = 0.640, RMSEA = 0.096), this indicates that the discriminative validity among the five variables in this study is good, which can be used for further data analysis.

**Table 1:** Discriminant validity test.

Model	Factor	χ^2^	*df*	χ^2^/*df*	TLI	CFI	GFI	NFI	RMSEA	∆χ^2^_(_∆*df*_)_
Five-factor model+CMF	DF,PD,SDF,LS,LO,CMF	2476.188	1,032	2.119	0.883	0.897	0.886	0.893	0.058	
Five-factor model	DF,PD,SDF,LS,LO	3154.414	1,081	2.918	0.840	0.852	0.841	0.847	0.068	
Four-factor model	DF + PD,SDF,LS,LO	3419.876	1,085	3.152	0.820	0.834	0.816	0.823	0.072	265.462***(4)
Three-factor model	DF + PD + SDF,LS,LO	4179.337	1,088	3.841	0.762	0.787	0.759	0.777	0.083	1024.923***(7)
Two-factor model	DF + PD + SDF + LS,LO	4804.200	1,090	4.408	0.715	0.736	0.711	0.726	0.091	1649.786***(9)
Single-factor model	DF + PD + SDF + LS + LO	5645.300	1,091	5.174	0.651	0.650	0.646	0.640	0.096	2499.886***(10)

*p < 0.05, **p < 0.01, ***p < 0.001. DF, Differential leadership; PD, Power distance; SDF, supervisor developmental feedback; LS, Loyalty to Supervisors; LO, Loyalty to Organizations, CMF, Common method factor, “+” stand for factor combination.

The effect of common method bias can be reduced through process control and statistical control. In this paper, privacy and confidentiality were emphasized in data collection to reduce the psychological concerns of the subjects, and the questionnaire was distributed at a series of different time to reduce the effect of common method bias through procedural control, but for the sake of scientific and rigorous research findings, the common method bias test was conducted by introducing the common method factor in the validation factor analysis The results are shown in Table 1, and the TLI, CFI, and RMSEA of the five-factor model were 0.840, 0.852 and 0.068, respectively. The TLI, CFI, and RMSEA in the five-factor model were 0.840, 0.852, and 0.068, respectively, and the TLI, CFI, and RMSEA were 0.883, 0.897, and 0.058 after the common method factor was added to the five-factor model, in which the change of TLI, CFI and RMSEA was less than 0.1 and the change of RMSEA was less than 0.05, indicating that the problem of common method bias in this paper was not serious.

### Descriptive statistics and correlation analysis

The means, standard deviations, correlation coefficients, and significance levels of each variable are shown in Table 2. Differential leadership was significantly and positively correlated with supervisor developmental feedback (*r* = 0.484, *p* < 0.001), power distance (*r* = 0.491, *p* < 0.001), organizational loyalty (*r* = 0.376, *p* < 0.001), and supervisory loyalty (*r* = 0.444, *p* < 0.001), and supervisor developmental feedback was also significantly and positively correlated with organizational loyalty (*r* = 0.390, *p* < 0.001)), supervisory loyalty (*r* = 0.693, *p* < 0.001), and power distance (*r* = 0.375, *p* < 0.001). The relationships between the variables were consistent with theoretical expectations and provided preliminary evidence for subsequent hypothesis testing.

**Table 2:** Descriptive statistics and correlation analysis.

Variable	M	SD	1	2	3	4	5	6	7	8	9	10
1. Age	2.93	0.88	1									
2. Gender:	1.30	0.46	−0.067	1								
3. WTL	2.45	1.13	0.171**	0.036	1							
4. WTO	2.48	1.13	0.192***	0.063	0.826***	1						
5. Rank	1.35	0.65	0.203***	−0.184***	0.339***	0.340***	1					
6. DF	4.83	1.13	0.057	−0.167**	0.065	0.085	0.053	1				
7. SDF	5.39	1.33	0.144**	−0.069	0.036	0.023	0.105*	0.484***	1			
8. PD	4.39	1.28	0.052	−0.071	0.085	0.097*	0.068	0.491***	0.375***	1		
9. LO	1.79	0.26	0.133**	−0.098*	0.130**	0.062	0.079	0.376***	0.390***	0.321***	1	
10. LS	5.70	0.88	0.199***	−0.016	0.138**	0.080	0.090	0.444***	0.693***	0.382***	0.615***	1

WTL, working time with direct leaders; WTO current organizational working time PD Power distance. *p < 0.05, **p < 0.01, ***p < 0.001.

### Test of hypotheses

SPSS22.0 was used for hierarchical regression analysis in this study to, respectively, test the hypotheses of the overall sample (412 individuals), the sample of insiders (249 individuals), and the sample of outsiders (163 individuals).

### Hypothesis testing based on 412 individuals

In this paper, a hierarchical linear regression model is used to test the direct effect and mediating effect proposed by the hypothesis, and the regression results are shown in Table 3. From M4 and M9, it can be known that differential leadership has a positive effect on employee loyalty, and the effect of “loyalty to supervisors” (*β* = 0.447，*p* < 0.001)is greater than that of “loyalty to organizations” (*β* = 0.366，*p* < 0.001) after controlling for the five demographic variables of age, gender, rank, working time with supervisors, and organizational working time. The H1 was supported. Then, the “three-step approach” is used to test for mediating effects. From M2, it can be known that differential leadership has a significantly positive effect on supervisor developmental feedback (*β* = 0.486，*p* < 0.001). Next, the variation of supervisor developmental feedback was added to the regression equation of differential leadership and organizational loyalty: It can be known from M5 that the regression coefficient of supervisor developmental feedback was 0.257 (*p* < 0.001), and the regression coefficient of differential leadership decreased to 0.241, but it was still significant at the level of 0.001, which indicated that supervisor developmental feedback had a partial mediating role in the relationship between differential leadership and organizational loyalty. Similarly, the variation of supervisor developmental feedback was added to the regression equation of differential leadership and supervisory loyalty: it can be known from M10 that the regression coefficient of supervisor developmental feedback was 0.607 (*p* < 0.001), and the regression coefficient of differential leadership decreased to 0.152, but it was also significant at the level of 0.001, which indicated that supervisor developmental feedback had a partial mediating role in the relationship between differential leadership and supervisory loyalty. The H2 was supported.

**Table 3:** Results of hierarchical regression analysis.

Variable	SDF		LO					LS				
	M1	M2	M3	M4	M5	M6	M7	M8	M9	M10	M11	M12
Age	−0.044	0.041	−0.088	−0.024	−0.034	−0.060	−0.042	0.003	0.081	0.056	0.041	0.055
Gender:	0.130*	0.115**	0.114*	0.103	0.073	0.070	0.078	0.184***	0.171***	0.101**	0.099**	0.105**
WTL	0.032	0.043	0.237**	0.245**	0.234**	0.225**	0.241**	0.214**	0.225**	0.198**	0.193**	0.205**
WTO	−0.052	−0.107	−0.153	−0.195*	−0.168*	−0.154	−0.180*	−0.142	−0.193*	−0.128*	−0.121	−0.141*
rank	0.078	0.085	0.011	0.017	−0.005	−0.016	−0.015	0.028	0.036	−0.016	−0.023	−0.022
DF		0.486***		0.366***	0.241***				0.447***	0.152***		
SDF					0.257***	0.299***	0.317***			0.607***	0.627***	0.641***
PD						0.198***	0.130*				0.142***	0.091*
PD × SDF							0.141**					0.108**
*F*	2.495	23.340	3.899	14.247	16.574	15.678	14.942	4.929	22.425	63.125	63.143	57.100
*R* ^2^	0.030	0.257	0.046	0.175	0.224	0.214	0.229	0.057	0.250	0.523	0.523	0.532
∆*R*^2^		0.227		0.129	0.049	0.045	0.015		0.193	0.273	0.017	0.009

*N* = 412, *p < 0.05, **p < 0.01, ***p < 0.001.

In order to make the results more rigorous, used Model 4 in Hayes' (2013) PROCESS macro with 5,000 bootstrap samples to test the mediation effect. The results are shown in Table 4. There are two hypothetical paths in organizational loyalty and supervisory loyalty (differential leadership → supervisor developmental feedback → organizational loyalty and differential leadership → supervisor developmental feedback → supervisory loyalty), and the confidence intervals are [0.014, 0.045] and [0.180, 0.287], which both do not contain “0,” and the indirect effect values are 0.029 and 0.230. After controlling for the mediating variables, the direct effects of differential leadership on organizational loyalty and leadership loyalty are still significant, and the 95% confidence intervals also do not contain “0,” which indicates that supervisor developmental feedback has a partially mediating role in differential leadership and organizational loyalty and supervisory loyalty. H2 is further supported.

**Table 4:** Results of mediation effect test.

Hypothetical paths	Effect	SE	Boot LL 95% CI	Boot UL 95% CI	Proportion
Direct effect: DF → LO	0.057	0.013	0.029	0.081	66%
Indirect effect: DF → SDF → LO	0.029	0.008	0.014	0.045	34%
Total effect	0.084	0.011	0.062	0.106	
Direct effect: DF → LS	0.119	0.039	0.041	0.195	34%
Indirect effec: DF → SDF → LS	0.230	0.027	0.180	0.287	66%
Total effect	0.349	0.039	0.270	0.425	

All coefficients are unstandardized. SE, standard error; LL, lower level; UL, upper level.

From M7 and M12 in Table 3 it can be known that the interaction effect of power distance and supervisor developmental feedback was significant (*β* = 0.141, *p* < 0.01) and (*β* = 0.108, *p* < 0.01), that is to say, power distance positively moderated the relationship between supervisor developmental feedback and organizational loyalty and supervisory loyalty. The significant interaction effect was further examined using simple slope analysis. When the dependent variable is loyalty to organizations, the association between power distance and supervisor developmental feedback was stronger for employees with high levels of power distance (simple slope = 0.075, *p* < 0.001) than for those with low levels of power distance (simple slope = 0.028, *p* < 0.005), and when the dependent variable is loyalty to supervisors, the association between power distance and supervisor developmental feedback was stronger for employees with high levels of power distance (simple slope = 0.471, *p* < 0.001) than for those with low levels of power distance (simple slope = 0.342, *p* < 0.001). H3 was supported.

In this paper, Model 14 in Hayes’ (2013) PROCESS macro was used to test the moderated mediating effect, and the results are shown in Table 5. For Loyalty to Organizations, when employees’ perceived power distance was low, the indirect effect of supervisor developmental feedback was not significant (Indirect effect = 0.016, 95% confidence interval = [−0.002, 0.034], including 0), when employees’ perceived power distance was high, the indirect effect of supervisor developmental feedback was significant (Indirect effect = 0.043, 95% confidence interval = [0.020, 0.072], excluding 0); for Loyalty to Supervisors, when employees’ perceived power distance was low, the indirect effect of supervisor developmental feedback was significant (Indirect effect = 0.031, 95% confidence interval = [0.138, 0.256], excluding 0), when employees’ perceived power distance perceptions were high (Indirect effect = 0.034, 95% confidence interval = [0.205, 0.337], excluding 0). It indicates that the mediating effect of being moderated is significant. H4 was supported.

**Table 5:** Results of moderated mediating effect.

	Loyalty to organizations				Loyalty to supervisors			
	Indirect effect	SE	95% confidence interval		Indirect effect	SE	95% confidence interval	
			LLCI	ULCI			LLCI	ULCI
M-1SD	0.016	0.009	−0.002	0.034	0.196	0.031	0.138	0.256
M	0.030	0.009	0.014	0.048	0.233	0.027	0.182	0.288
M + 1SD	0.043	0.013	0.020	0.072	0.270	0.034	0.205	0.337

### Hypothesis testing based on 249 individuals (insiders)

In this paper, a hierarchical linear regression model is used to test the direct effect and mediating effect proposed by the hypothesis, and the regression results are shown in Table 6. From M4 and M9, it can be known that differential leadership has a positive effect on employee loyalty, and the effect of “loyalty to supervisors” (*β* = 0.550, *p* < 0.001)is greater than that of “loyalty to organizations”(*β* = 0.386, *p* < 0.001) after controlling for the five demographic variables of age, gender, rank, working time with supervisors, and organizational working time. The H1 was supported.

**Table 6:** Results of hierarchical regression analysis.

Variable	SDF		LO					LS				
	M1	M2	M3	M4	M5	M6	M7	M8	M9	M10	M11	M12
Age	−0.076	0.069	−0.165*	−0.052	−0.064	−0.105	−0.102	−0.081	0.081	0.046	−0.004	−0.003
Gender:	−0.024	0.042	−0.068	−0.017	−0.024	−0.042	−0.035	−0.016	0.057	0.036	0.013	0.017
WTL	−0.121	−0.112	0.215	0.222*	0.241*	0.262*	0.272*	0.170	0.181	0.237**	0.255**	0.261**
WTO	0.176	0.080	−0.145	−0.220*	−0.233*	−0.233*	−0.260*	−0.007	−0.114	−0.154*	−0.146	−0.160
5 rank	0.126	0.124	0.098	0.097	0.076	0.071	0.072	0.052	0.051	−0.012	−0.020	−0.019
DF		0.495***		0.386***	0.303***				0.550***	0.299***		
SDF					0.168**	0.237***	0.266***			0.507***	0.591***	0.607***
PD						0.240***	0.163*				0.179***	0.137*
PD × SDF							0.208**					0.111*
*F*	1.823	13.704	3.312	9.815	9.528	8.836	9.536	2.121	18.133	34.655	30.300	27.550
*R* ^2^	0.036	0.235	0.064	0.196	0.217	0.204	0.241	0.042	0.310	0.502	0.468	0.479
∆*R*^2^		0.199		0.132	0.021	0.05	0.037		0.268	0.192	0.028	0.011

*N* = 249, *p < 0.05, **p < 0.01, ***p < 0.001.

Then, the “three-step approach” is used to test for mediating effects. From M2, it can be known that differential leadership has a significantly positive effect on supervisor developmental feedback (*β* = 0.495, *p* < 0.001). Next, the variation of supervisor developmental feedback was added to the regression equation of differential leadership and organizational loyalty: It can be known from M5 that the regression coefficient of supervisor developmental feedback was 0.168 (*p* < 0.001), and the regression coefficient of differential leadership decreased to 0.303, but it was still significant at the level of 0.001, which indicated that supervisor developmental feedback had a partial mediating role in the relationship between differential leadership and organizational loyalty; Similarly, the variation of supervisor developmental feedback was added to the regression equation of differential leadership and supervisory loyalty: it can be known from M10 that the regression coefficient of supervisor developmental feedback was 0.507 (*p* < 0.001), and the regression coefficient of differential leadership decreased to 0.299, but it was also significant at the level of 0.001, which indicated that supervisor developmental feedback had a partial mediating role in the relationship between differential leadership and supervisory loyalty. The H2 was supported.

In order to make the results more rigorous, used Model 4 in Hayes’ (2013) PROCESS macro with 5,000 bootstrap samples to test the mediation effect. The results are shown in Table 7. There are two hypothetical paths in organizational loyalty and supervisory loyalty (differential leadership → supervisor developmental feedback → organizational loyalty and differential leadership → supervisor developmental feedback → supervisory loyalty), and the confidence intervals are [0.003, 0.035] and [0.111, 0.236], which both do not contain “0,” and the indirect effect values are 0.018 and 0.167. After controlling for the mediating variables, the direct effects of differential leadership on organizational loyalty and leadership loyalty are still significant, and the 95% confidence intervals also do not contain “0,” which indicates that supervisor developmental feedback has a partially mediating role in differential leadership and organizational loyalty and supervisory loyalty. H3 is further supported.

**Table 7:** Results of mediation effect test.

hypothetical paths	Effect	SE	Boot LL 95% CI	Boot UL 95% CI	Proportion
Direct effect: DF → LO	0.065	0.015	0.036	0.094	79%
Indirect effect: DF → SDF → LO	0.018	0.008	0.003	0.035	21%
Total effect	0.083	0.013	0.059	0.108	
Direct effect: DF → LS	0.199	0.038	0.124	0.273	54%
Indirect effect: DF → SDF → LS	0.167	0.032	0.111	0.236	46%
Total effect	0.365	0.044	0.280	0.451	

All coefficients are unstandardized. SE, standard error; LL, lower level; UL, upper level.

From M7 and M12 in Table 6 it can be known that the interaction effect of power distance and supervisor developmental feedback was significant (*β* = 0.208, *p* < 0.01) and (*β* = 0.111, *p* < 0.05), that is to say, power distance positively moderated the relationship between supervisor developmental feedback and organizational loyalty and supervisory loyalty. The significant interaction effect was further examined using simple slope analysis. When the dependent variable is loyalty to organizations, the association between power distance and supervisor developmental feedback was stronger for employees with high levels of power distance (simple slope = 0.103, *p* < 0.001) than for those with low levels of power distance (simple slope = 0.052, *p* < 0.01), and When the dependent variable is loyalty to supervisors, the association between power distance and supervisor developmental feedback was stronger for employees with high levels of power distance (simple slope = 0.452, *p* < 0.001) than for those with low levels of power distance (simple slope = 0.322, *p* < 0.001). H3 was supported.

In this paper, Model 14 in Hayes’ (2013) PROCESS macro was used to test the moderated mediating effect, and the results are shown in Table 8. For Loyalty to Organizations, when employees’ perceived power distance was low, the indirect effect of supervisor developmental feedback was not significant (Indirect effect = 0.055, 95% confidence interval = [−0.012, 0.021], including 0), when employees’ perceived power distance was high, the indirect effect of supervisor developmental feedback was significant (Indirect effect = 0.048, 95% confidence interval = [0.016, 0.087], excluding 0); for Loyalty to Supervisors, when employees’ perceived power distance was low, the indirect effect of supervisor developmental feedback was significant (Indirect effect = 0.149 95% confidence interval = [0.083, 0.232], excluding 0), when employees’ perceived power distance perceptions were high (Indirect effect = 0.209, 95% confidence interval = [0.126,0.301], excluding 0). It indicates that the mediating effect of being moderated is significant. H4 was supported.

**Table 8:** Results of moderated mediating effect.

	Loyalty to organizations				Loyalty to supervisors			
	Indirect effect	SE	95% confidence interval		Indirect effect	SE	95% confidence interval	
			LLCI	ULCI			LLCI	ULCI
M−1SD	0.055	0.082	−0.012	0.021	0.149	0.039	0.083	0.232
*M*	0.027	0.011	0.007	0.049	0.179	0.033	0.119	0.250
*M* + 1SD	0.048	0.018	0.016	0.087	0.209	0.045	0.126	0.301

### Hypothesis testing based on 163 individuals (outsiders)

In this paper, a hierarchical linear regression model is used to test the direct effect and mediating effect proposed by the hypothesis, and the regression results are shown in Table 9. From M4 and M9, it can be known that differential leadership has a positive effect on employee loyalty, and the effect of “loyalty to supervisors” (*β* = 0.246，*p* < 0.001)is greater than that of “loyalty to organizations”(*β* = 0.388，*p* < 0.001) after controlling for the five demographic variables of age, gender, rank, working time with supervisors, and organizational working time. The H1 was supported. Then, the “three-step approach” is used to test for mediating effects. From M2, it can be known that differential leadership has a significantly positive effect on supervisor developmental feedback (*β* = 0.366，*p* < 0.001). Next, the variation of supervisor developmental feedback was added to the regression equation of differential leadership and organizational loyalty: It can be known from M5 that the regression coefficient of supervisor developmental feedback was0.569(*p* < 0.001), and the regression coefficient of differential leadership decreased to 0.180, but it was still significant at the level of 0.001, which indicated that supervisor developmental feedback had a partial mediating role in the relationship between differential leadership and organizational loyalty. Similarly, the variation of supervisor developmental feedback was added to the regression equation of differential leadership and supervisory loyalty: it can be known from M10 that the regression coefficient of supervisor developmental feedback was 0.650 (*p* < 0.001), and the regression coefficient of differential leadership decreased to 0.008, which is no longer significant, which indicates that superior developmental feedback plays a completely mediating role in the relationship between differential leadership and loyalty to supervisors, that is, the effect of differential leadership on loyalty to supervisors is completely realized through superior developmental feedback. The H2 was supported.

**Table 9:** Results of hierarchical regression analysis.

Variable	SDF		LO					LS				
	M1	M2	M3	M4	M5	M6	M7	M8	M9	M10	M11	M12
Age	0.115*	0.096*	0.162**	0.142**	0.087*	0.079*	0.084*	0.193***	0.780***	0.118**	0.115**	0.116***
Gender:	−0.013	0.048	−0.040	0.025	−0.003	−0.020	−0.011	0.037	0.079	0.047	0.09	0.049
WTL	0.059	0.064	0.121	0.127	0.090	0.087	0.107*	0.130	0.134	0.092	0.092	0.094
WTO	−0.089	−0.123	−0.090	−0.125	−0.056	−0.059	−0.081	−0.077	−0.099	−0.020	−0.024	−0.026
5rank	0.109*	0.110*	0.043	0.044	−0.019	−0.019	−0.020	0.028	0.029	−0.042	−0.041	−0.041
DF		0.366***		0.388***	0.180***				0.246***	0.008		
SDF					0.569***	0.560***	0.588***			0.650***	0.635***	0.637***
PD						0.286***	0.229***				0.067	0.064
PD × SDF							0.174***					0.010
*F*	2.995	14.838	4.104	17.893	36.702	68.476	66.533	5.009	9.521	57.575	58.522	51.114
*R* ^2^	0.031	0.160	0.042	0.187	0.458	0.507	0.533	0.051	0.109	0.463	0.467	0.467
∆*R*^2^		0.129		0.145	0.271	0.076	0.026		0.058	0.354	0.004	0.000

*N* = 163, *p < 0.05, **p < 0.01, ***p < 0.001.

In order to make the results more rigorous, used Model 4 in Hayes’ (2013) PROCESS macro with 5,000 bootstrap samples to test the mediation effect. The results are shown in Table 10. The confidence interval of differential leadership → superior developmental feedback → organizational loyalty is [0.123, 0.220], excluding 0, and the indirect effect value is 0.171. After controlling the intermediary variable, the direct effect of differential leadership on organizational loyalty is still obvious, and 95% confidence interval does not contain 0, indicating that the superior developmental feedback has a partial intermediary role in differential leadership and organizational loyalty and leadership loyalty. The confidence interval of the path of differential leadership → superior developmental feedback → supervisory loyalty is [0.121, 0.226], which does not contain 0, and the indirect effect value is 0.173. After controlling the mediating variable, the direct effect of differential leadership on leadership loyalty is not obvious, and 95% confidence interval contains 0. It shows that the superior developmental feedback has a complete mediating effect between differential leadership and leadership loyalty, and H2 is further supported.

**Table 10:** Results of mediation effect test.

hypothetical paths	Effect	SE	Boot LL 95% CI	Boot UL 95% CI	Proportion
Direct effect: DF → LO	0.148	0.038	0.078	0.226	46%
Indirect effect: DF → SDF → LO	0.171	0.025	0.123	0.220	54%
Total effect	0.319	0.047	0.228	0.412	
Direct effect: DF → LS	0.006	0.032	−0.057	0.069	3%
Indirect effect: DF → SDF → LS	0.173	0.027	0.121	0.226	97%
Total effect					

All coefficients are unstandardized. SE, standard error; LL, lower level; UL, upper level.

From M7 and M12 in Table 9 it can be known that, for loyalty to organizations, the interaction effect of power distance and supervisor developmental feedback was significant (*β* = 0.174, *p* < 0.001). That is to say, power distance positively moderated the relationship between supervisor developmental feedback and organizational loyalty. But for loyalty to supervisors, the interaction effect was not significant (*β* = 0.010, *p* > 0.05). The significant interaction effect was further examined using simple slope analysis, the association between power distance and supervisor developmental feedback was stronger for employees with high levels of power distance (simple slope = 0.554, *p* < 0.001) than for those with low levels of power distance (simple slope = 0.316, *p* < 0.01). H3 was partially supported.

In this paper, Model 14 in Hayes' (2013) PROCESS macro was used to test the moderated mediating effect, and the results are shown in Table 11. For organizational loyalty, when employees’ perceived power distance was low, the indirect effect of supervisor developmental feedback was significant (Indirect effect = 0.128, 95% confidence interval = [0.081, 0.180], excluding 0), when employees’ perceived power distance was high, the indirect effect of supervisor developmental feedback was significant (Indirect effect = 0.221, 95% confidence interval = [0.158, 0.290], excluding 0). H4 was partially supported.

**Table 11:** Results of moderated mediating effect test.

	Loyalty to organizations			
	Indirect effect	SE	95% confidence interval	
			LLCI	LLCI
*M*−1SD	0.128	0.026	0.081	0.180
*M*	0.174	0.025	0.127	0.223
*M* + 1SD	0.221	0.034	0.158	0.290

## Discussion

Unlike previous studies that focused on the relationship between differential leadership and organizational citizenship behavior, this paper focuses on the types of employee loyalty and constructs a theoretical model of differential leadership and organizational loyalty and leadership loyalty in the context of Chinese culture, and finally conducts an empirical analysis from a perspective of insider and outsider on the basis of social exchange theory and social learning theory. Research indicates:

First, differential leadership has a positive effect on employees’ organizational loyalty and supervisory loyalty. No matter in the overall samples or the sample of insiders, the positive effect of differential leadership on employees’ supervisory loyalty is significantly higher than that of differential leadership on organizational loyalty. However, in the samples of outsiders, the positive effect of differential leadership on employees’ supervisory loyalty was lower than that on organizational loyalty.

Second, the effects of differential leadership on supervisory loyalty of insiders and outsiders are significantly different. Specifically, the regression coefficient of differential leadership on supervisory loyalty of outsiders is 0.246 and that of differential leadership on supervisory loyalty of insiders is 0.550, but the disparity between the effects of differential leadership on organizational loyalty from insiders and outsiders is not significant.

Third, supervisory developmental feedback mediates the relationship between differential leadership and organizational and supervisory loyalty.

Fourth, power distance positively moderates the relationship between supervisory developmental feedback and organizational and leadership loyalty, and the higher the employee perceives power distance, the stronger the indirect effect of supervisor developmental feedback on employees’ organizational and supervisory loyalty is.

### Theoretical implications

First, differential leadership, as a common and unique leadership style in Chinese organizations, has not been paid much attention by academia. This study confirms the relationship between differential leadership and employee loyalty. The positive influence of differential leadership on employee loyalty could be explained from the two aspects of “reciprocal” (bao) theory and social learning theory. On the one hand, the “reciprocal” (bao) culture has a profound historical origin in the Chinese traditional culture, and “return a favor with a favor” has always been the mainstream value respected by the Chinese nation, so when the insiders receive the partiality from differential leadership (e.g., insiders will get rewards and quick promotions when they do well in their positions, insiders will receive encouragement and guidance when they make mistakes.), they make good psychological contracts and show great loyalty to their supervisors; on the other hand, the partiality from differential leadership is much like a booster for employee’s career development and life goals and is equally important for outsiders. So if the outsider wants to receive the same treatment, they must become an insider from an outsider, according to the alternative reinforcement mechanism of the social learning theory, loyalty to supervisors is one of the best ways. In the three-way game between the leader and the insider and the outsider, the insiders will show greater loyalty to the supervisor because they are favored; while the outsiders will want to become an insider because they are not favored. This game process continuously strengthens the cohesion and radiation of the “influence,” and also enhances employee loyalty. This study selects the differential leadership with local cultural adaptability and characteristics as a breakthrough, which not only enriches the theory of local leadership but also is a useful supplement to the influencing factors of loyalty behavior.

Second, the study on the influence of differential leadership and loyalty behavior from a perspective of insiders and outsiders broke the logical limitations of previous studies from a single perspective and conducted separate tests for the samples of insiders and outsiders as well as the overall test, which not only improved the accuracy of the test results but also provided a detailed data comparison for the differences of the results. In the first place, it was found that the regression coefficient of supervisory loyalty was significantly larger than that of organizational loyalty in the sample of insiders, that is to say, differential leadership has a more significant effect on the formation of “loyalty to supervisors,” but in the sample outsiders, the regression coefficient of leadership loyalty was smaller than that of organizational loyalty, which may be due to the fact that outsiders lack of psychological attachment to their direct supervisors compared with the insiders. They only maintain an instrumental relationship with their leaders that only involves the equal exchange of benefits and harms (no loss is the goal). The lower level of emotional exchange between employees and supervisors makes outsiders strongly believe that there is no significant difference in the concept and connotation of “organization” and “leadership,” and unconsciously regard the supervisor developmental feedback as care from the organization, instead of a sort of partiality from supervisors. Therefore, the regression coefficient of employee loyalty to the organization of outsider is slightly higher than that of loyalty to supervisors; next, It can be found that the regression coefficients of differential leadership on the formation of supervisory loyalty of insiders is much higher than that of outsiders, that is to say, employee loyalty to supervisory of insiders is significantly obvious than that of outsiders. This is consistent with the multifocal perspective and the object consistency model in organizational behavior research: the receiver’s response is directly dependent on the specific object that releases the signal. After differential leadership releases a signal of partiality, it stimulates insiders to show positive behaviors of loyalty to the supervisor. Although the signal also stimulates outsiders to become insiders by showing their loyalty to the supervisor, the degree of loyalty of outsiders is definitely not as high as that of insiders. This study examines differential leadership and loyalty behavior from a perspective of insider and outsider, which is not only supplement and extension of existing single perspective studies but also provides empirical support for understanding the differences in cognitive behavior between insiders and outsiders.

Third, this study further reveals the process mechanism of the influence of differential leadership on employee loyalty, verifies the mediating role of supervisor developmental feedback, analyzes an important inducing factor of employee loyalty, and to some extent exposes the “black box” (an intermediate mechanism) of differential leadership influence on employee loyalty.

Fourth, from the perspective of employee traits, this study examines the moderating role of power distance in the formation of employee loyalty, and supplements the boundary conditions for employee loyalty behavior. As a country with a high power distance, people in China generally respect authority and are very sensitive to the behavior of authority figures. Employees who live in a high power culture are more likely to return a psychological favor after receiving developmental feedback from their supervisors and then behave in a loyal way to their supervisors and organizations. This suggests that the formation of employee loyalty not only depends on leadership style but also on employee traits to some extent, so the two factors cannot be generalized and confusing. This study selects power distance as a moderator when examining the mechanism of differential leadership on employee loyalty, and finds that outsiders feel a sense of supervisor unfairness and organization alienation, the result is that outsiders could not make the psychological contract with their supervisors. Therefore, outsiders have unclearer thoughts of the organization and their immediate supervisor than insiders, and even generally regard the two as the same concept. So the moderating effect of power distance on the formation of leadership loyalty of outsiders is not obvious, but the moderating effect in other samples is significant. This study examines the moderating effect from a perspective of insiders and outsiders, further clarifies the boundary conditions for differential leadership to be effective under different conditions, and provides a more detailed classification for the formation of loyalty behavior.

### Practical implications

First of all, leaders should take emphasis on their own management style and provide a fair and reasonable channel for outsiders to become an insider. While giving support and feedback to the insider, leaders should also actively communicate with outsiders, create a harmonious organizational atmosphere, improve the relationship between superiors and subordinates, in order to attract more outsiders, maximize the effectiveness of leadership, and constantly improve the overall loyalty of the organization.

Secondly, leaders can actively implement the strategy of supervisor developmental feedback, which is the intermediate path to stimulate and promote employee loyalty behavior. Leaders should give full play to their initiative and actively communicate this feedback to employees in an appropriate manner and with the right frequency. At the same time, they should enhance their learning on feedback and improve its way and quality so that Employees could feel the care and partiality of their supervisors, thus improving employee loyalty.

Finally, Perceived power distance of employees could moderate the effect of differential leadership on employees’ loyalty behavior. In the practical management process, leaders should implement differential management according to the level of perceived power distance of employees. Especially for those employees with a high power distance, they are more likely to return psychological favor after receiving positive feedback from supervisors. Therefore, leaders should pay more attention to communicating with those high power distance employees reasonably and effectively in order to maximize their employee loyalty.

### Limitations and future research

Although this paper uses a multi-period method to distribute the questionnaires, since the questionnaires are filled out by the participants in the form of self-assessment, even if the problem of common method bias is not serious, the problem cannot be completely eliminated. In the future, the sources of other evaluation data should be increased to reduce the common method bias. In addition, this study confirmed that differential leadership has a positive effect on employee loyalty, but in general, such loyalty can be classified as “loyalty to supervisors.” However, on the individual level, there is no doubt that loyalty to supervisors could reduce and avoid conflicts between superiors and subordinates and thereby improve the management efficiency of the leader, but at the organization-wide level, overemphasizing leadership loyalty will cause factions within the organization, which in turn affects the overall efficiency of the organization. Therefore, future research should further explore how to achieve the integration of “loyalty to organizations” and “loyalty to supervisors” in order to maximize the overall effectiveness of the organization.

## Data availability statement

The raw data supporting the conclusions of this article will be made available by the authors, without undue reservation.

## Ethics statement

The studies involving human participants were reviewed and approved by the Ethics Committee of Yunnan University of Finance and Economics. The patients/participants provided their written informed consent to participate in this study.

## Author contributions

HZ: supervision and funding acquisition. LD: conceptualization, methodology, formal analysis, and writing—original draft. ZJ: editing. All authors contributed to the article and approved the submitted version.

## Funding

This study is supported by the National Natural Science Foundation of China “Research on the Causes, Evolution and Resolution Mechanism of Ought to be Conflicts in Mixed Ownership Reform: The Perspective of mutual beneficial organization”. (Grant No. 72262035).

## Conflict of interest

The authors declare that the research was conducted in the absence of any commercial or financial relationships that could be construed as a potential conflict of interest.

## Publisher’s note

All claims expressed in this article are solely those of the authors and do not necessarily represent those of their affiliated organizations, or those of the publisher, the editors and the reviewers. Any product that may be evaluated in this article, or claim that may be made by its manufacturer, is not guaranteed or endorsed by the publisher.
